# Switchable Lipid Provides pH-Sensitive Properties to Lipid and Hybrid Polymer/Lipid Membranes

**DOI:** 10.3390/polym12030637

**Published:** 2020-03-11

**Authors:** Victor Passos Gibson, Martin Fauquignon, Emmanuel Ibarboure, Jeanne Leblond Chain, Jean-François Le Meins

**Affiliations:** 1 Gene Delivery Laboratory, Faculty of pharmacy, University of Montréal, Montréal, QC H3C 3J7, Canada; victor.passos.gibson@umontreal.ca; 2 Laboratoire de Chimie des Polymères Organiques, LCPO, Université de Bordeaux, CNRS, Bordeaux INP, UMR 5629, Avenue Pey Berland, F-33600 Pessac, France; Martin.Fauquignon@enscbp.fr (M.F.); Emmanuel.Ibarboure@enscbp.fr (E.I.); 3 ARNA Laboratory, INSERM U1212, CNRS UMR 5320, University of Bordeaux, Faculty of pharmacy, F-33016 Bordeaux, France

**Keywords:** hybrid polymer/lipid membrane, pH-sensitive liposomes, switchable lipid, giant unilamellar vesicles, giant hybrid polymer/lipid vesicles, responsive vesicles

## Abstract

Blending amphiphilic copolymers and lipids constitutes a novel approach to combine the advantages of polymersomes and liposomes into a new single hybrid membrane. Efforts have been made to design stimuli-responsive vesicles, in which the membrane’s dynamic is modulated by specific triggers. In this investigation, we proposed the design of pH-responsive hybrid vesicles formulated with poly(dimethylsiloxane)-*block*-poly(ethylene oxide) backbone (PDMS_36_-*b*-PEO_23_) and cationic switchable lipid (CSL). The latter undergoes a pH-triggered conformational change and induces membrane destabilization. Using confocal imaging and DLS measurements, we interrogated the structural changes in CSL-doped lipid and hybrid polymer/lipid unilamellar vesicles at the micro- and nanometric scale, respectively. Both switchable giant unilamellar lipid vesicles (GUV) and hybrid polymer/lipid unilamellar vesicles (GHUV) presented dynamic morphological changes, including protrusions and fission upon acidification. At the submicron scale, scattered intensity decreased for both switchable large unilamellar vesicles (LUV) and hybrid vesicles (LHUV) under acidic pH. Finally, monitoring the fluorescence leakage of encapsulated calcein, we attested that CSL increased the permeability of GUV and GHUV in a pH-specific fashion. Altogether, these results show that switchable lipids provide a pH-sensitive behavior to hybrid polymer/lipid vesicles that could be exploited for the triggered release of drugs, cell biomimicry studies, or as bioinspired micro/nanoreactors.

## 1. Introduction

Liposomes were initially developed as simplified cell membrane analogs and rapidly recognized as promising vesicles for a wide range of pharmaceutical applications, especially as drug delivery carriers [1]. More than 15 liposomal-based drug formulations have reached the market [2] (e.g., AmBisome^®^, Doxil^®^/Caelyx^®^, and DepoCyt^®^), and, recently, lipid nanoparticles enabled the delivery of the first RNAi-based drug (OnPattro^®^), which was a milestone for gene therapy [3]. Nonetheless, liposomes still suffer from short shelf life due to their low stability and poor control over membrane leakage. On the other end, polymersomes have been developed to overcome these limitations. Similar to liposomes, such vesicles result from the self-assembly of amphiphilic copolymers, exhibiting more robust properties, such as a more stable and less permeable membrane [4].

Recently, it became possible to combine the advantages of both polymersomes and liposomes into a new single hybrid unilamellar vesicle. Hybrid polymer/lipid vesicles combine the stiffness and stability of polymersomes with the biocompatibility and chemical functionality of phospholipids [5]. Modulation of the structuration of the hybrid membrane is possible by playing with the nature of polymer and lipids used and the polymer-to-lipid molar ratio [6]. However, the membrane permeability remains an issue, as passive diffusion or membrane disruption are the main methods of payload release [7]. Therefore, efforts have been made to formulate “smart” hybrid polymer/lipid vesicles, which would be able to change their membrane properties in response to specific triggers [8]. In particular, pH-sensitive vesicles allow specific drug delivery at acidic conditions encountered in some pathological microenvironments, e.g., cancer [9], inflammation [10], and ischemia [11,12], or upon vesicle endocytosis in early endosomes [13]. We have recently developed pH-sensitive lipids that undergo a conformational switch at acidic pH [14]. Incorporated into lipid nanoparticles, they destabilize the lipid membrane in a pH-responsive fashion through a mechanism involving fusion. Thanks to their endosomal escape ability, they massively release their cargo in less than 30 min, resulting in good in vitro and in vivo transfection of siRNA, for cancer [15], and hypercholesterolemia applications [16].

In polymersomes, stimuli-responsive materials have been explored thanks to the versatility of polymer chemistry [7]. Chen et al. formulated pH-sensitive polymersomes based on a poly(ethylene glycol) (PEG) moiety-grafted acid-labile polycarbonate able to encapsulate both paclitaxel and doxorubicin hydrochloride (DOX.HCl) and promote their release upon acid-triggered hydrolysis [17]. Similarly, Liu et al. encapsulated DOX and DOX.HCl in pH-sensitive polymersomes based on poly(D,L-lactide)-*block*-poly(2-methacryloyloxyethyl phosphorylcholine) [18]. However, in both cases, only partial drug release was obtained after 24 h of incubation. Quicker responding systems would be desired to trigger burst drug release at the target site or to match the time course of endosomal maturation, which is under 1 h [19].

In this study, we hypothesized that doping polymeric membranes composed of poly(dimethylsiloxane)-*block*-poly(ethylene oxide) (PDMS-*b*-PEO) with switchable lipids would provide pH-sensitive properties to the resulting hybrid polymer/lipid vesicles. PDMS-*b*-PEO was selected because both blocks are biocompatible and PDMS exhibits flexible chains, which is required to prepare giant vesicle by the hydration process. In addition, such PDMS-*b*-PEO polymersomes have demonstrated their ability to form stable hybrid edifices [20]. We exploited the development of giant unilamellar vesicles [21] to investigate the dynamic behavior of switchable lipids in either lipid or hybrid polymer/lipid unilamellar membranes (GUV and GHUV) at an acidic environment using confocal imaging. We compared the results with dynamic light scattering (DLS) measurements of large unilamellar lipid or hybrid polymer/lipid vesicles [21] (LUV and LHUV) at acidic pH. Lastly, we analyzed, qualitatively and quantitatively, the influence of switchable lipids on membrane’s permeability of calcein-loaded GUV and GHUV in an acidic microenvironment.

## 2. Materials and Methods

All organics solvents and chemicals were purchased from Sigma-Aldrich (Sigma-Aldrich Chimie, Saint Quentin Fallavier, France) and Thermo Scientific (Waltham, MA, USA). 1-Palmitoyl-2-oleoyl-sn-glycero-3-phosphocholine (POPC) and 1,2-dioleoyl-sn-glycero-3-phosphoethanolamine-N-(lissamine rhodamine B sulfonyl) (Rhodamine-PE) were obtained from Avanti Polar Lipids Inc. (Alabaster, AL, USA). Poly(dimethylsiloxane)-nitrobenzoxadiazole (PDMS-NBD) and poly(dimethylsiloxane)-*block*-poly(ethylene oxide) (PDMS_36_-*b*-PEO_23_, M_n_ 4000 g.mol^-1^) were synthesized as previously described [20]. This copolymer is known to spontaneously form polymersomes with a membrane thickness of 9.9 ± 1.6 nm. Cationic switchable lipid (CSL) was synthesized as previously described [16]. Sucrose, calcein and all other chemicals were purchased from Sigma-Aldrich (Saint Quentin Fallavier, France). ^1^H Nuclear Magnetic Resonance spectra were acquired at 298 K on a Bruker Avance I NMR spectrometer operating at 400 MHz, using trimethylsilane (TMS) as the internal standard and deuterated chloroform as solvent. Data were treated with TopSpin 4.0.7 (Bruker BioSpin, Wissembourg, France).

### 2.1. GUV and GHUV Preparation

Giant unilamellar vesicles (GUV) and giant hybrid unilamellar vesicles (GHUV) were prepared by the electroformation method proposed by Angela et al. [22]. Briefly, a 1.4 mg.mL^−1^ solution of lipids (POPC or a mixture of POPC:CSL at a ratio 80:20 or 50:50 mol %) in chloroform or a mixture of polymer (PDMS_36_-*b*-PEO_23_) and lipid (POPC or CSL) at specific ratio (polymer/lipid 80:20 or 50:50 mol %) was deposited thrice in each side of an indium tin oxide (ITO)-coated glass slide (Sigma Aldrich, Saint Quentin Fallavier, France) using a capillary tip. For confocal visualization, two probes were added to the appropriate lipid or polymer/lipid organic solution: rhodamine-PE at 0.1% *w/w* for lipid bilayer visualization and/or PDMS-NBD at 1% *w/w* for polymer visualization. The electroformation chamber was set by connecting both ITO-covered slides using a rubber O-ring, which were then dried under vacuum overnight. The next day, samples were connected to an AC generator and an alternative voltage (10 Hz, 2 V) was applied, followed by the immediate addition of 200 μL of 100 mM sucrose solution. GUV or GHUV were collected after 45 min of electroformation using a syringe with 21-gauge needle.

To study the effect of acidic conditions onto GUV and GHUV membranes’ permeability, giant vesicles were hydrated with 100 μL of 100 mM sucrose solution containing 10 μM of calcein during electroformation. GUV and GHUV were harvested after 45 min of electroformation using a syringe with a 21-gauge needle. Non-encapsulated calcein was removed by dialysis against sucrose 100 mM overnight at room temperature using a Float-A-Lyzer G2 device (MWCO 50 KDa, Sigma-Aldrich, Saint Quentin Fallavier, France). Finally, calcein-loaded GUV and GHUV were harvested in a 0.5 mL dark Eppendorf and protected from light until testing. Samples were analyzed within 24 h after preparation.

### 2.2. LUV and LHUV Preparation

Large unilamellar vesicles (LUV) and large hybrid unilamellar vesicles (LHUV) were prepared using the lipid film hydration method. A lipid or a polymer/lipid solution in chloroform at the desired ratio (for LUV, 80:20 or 50:50 mol % POPC:CSL; for GHUV, 80:20 or 50:50 mol % PDMS_36_-*b*-PEO_23_:CSL) was added in a 25 mL round-bottom flask, dried under reduced pression in a rotary evaporator, and further dried under vacuum for at least 2 h before hydration with 10 mL of ultrapure water at room temperature without any agitation to yield a 1.4 mg mL^−1^ lipid suspension. Afterwards, the suspension was extruded 11 times through a 1 μm polycarbonate membrane (Avanti Polar Lipids Inc., Alabaster, AL, USA) for DLS measurement.

### 2.3. ^1^H NMR Measurement

For ^1^H NMR measurements only, GUV or GHUV were prepared by the hydration of the lipid film as mentioned above for LUV and LHUV, in order to get the sufficient amount of particles. After hydration, vesicles were not extruded but rather transferred to another 150 mL round-bottom flask for further freeze-drying. Then, the powder was resuspended in 500 μL of deuterated chloroform for ^1^H NMR analysis to reach a final CSL concentration of 7 mg mL^−1^. Other components were calculated according to the POPC:CSL ratio, in GUV, or PDMS_36_-*b*-PEO_23_:CSL in GHUV. Samples were analyzed the same day as preparation.

### 2.4. Dynamic Light Scattering 

Hydrodynamic diameter and ζ-potential of LUV or LHUV suspension were measured at 20 °C using a Malvern Zetasizer Nano ZS (Malvern, Worcestershire, UK). Samples were diluted in Milli-Q water (1:2 *v/v*) to a final volume of 1 mL. Size measurements were performed with a scattered angle of 173° and reported as Z-average (intensity). The voltage for ζ-potential was set automatically by the equipment. In order to evaluate the effect of pH change on the LUV or LHUV properties, samples were acidified with HCl 0.01 M and monitored using a pH-meter until a drop of pH from 6.8 to 2.8 was reached. When applicable, an equivalent amount to HCl of NaCl at the same molarity was added to vesicles suspension as a control. Measurements were performed at least in triplicate. Graphs were plotted using GraphPad (Prism 7, GraphPad Software Inc., San Diego, CA, USA).

### 2.5. Confocal Imaging

All images were acquired on a Leica TCS SP5 (Leica Microsystems CMS GmbH, Mannheim, Germany) inverted confocal microscope (DMI6000). A 50-μL aliquot of 100 mM sucrose suspension of GUV or GHUV was added into 150 μL iso-osmolar (100 mOsm L^−1^) glucose solution containing in an eight-well μ-Slide (Ibidi, Martinsried, Germany). Vesicles were allowed to sediment for at least 2 min before imaging. It is important to stress that vesicles were formulated in sucrose 100 mM and allowed to sediment in a glucose solution prepared at same concentration to avoid osmolarity shock. In order to evaluate the morphological and/or loaded-calcein intensity changes upon acidification, 39 μL of an iso-osmolar HCl solution in glucose (100 mOsm L^−1^, 4 × 10^−4^ mM) was added directly in the well chamber. This volume of HCl was sufficient to, within 2 min, drop the pH down to 4.7, which was a value lower than the pH of the CSL conformation switch [16]. As a control, the same volume of an iso-osmolar sucrose solution of NaCl was added to the vesicles-containing chamber. PDMS-NBD/calcein and rhodamine-PE were stepwisely imaged using an argon laser line with an excitation/range of emission of 488 nm/500–530 nm (PDMS-NBD/calcein) and 514 nm/600–700 nm (Rhodamine-PE). Images were processed using Fiji/ImageJ software.

To calculate the overall fluorescence intensity of loaded calcein immediately before and after (2 min) HCl treatment, 13 images per condition were processed by ImageJ software on the same Region of Interest (ROI). Fluorescence intensity ranged from 0 to 250 and the number of pixels for each intensity was normalized by the total amount of pixels measured. A normal distribution of fluorescence intensity was plotted using GraphPad (Prism 7, GraphPad Software Inc., San Diego, CA, USA).

## 3. Results

### 3.1. Insertion of CSL into LUV or LHUV

In order to assess the incorporation of cationic switchable lipid (CSL) in lipid and hybrid polymer/lipid membranes, we prepared a series of vesicles and assessed their hydrodynamic diameter and polydispersity index (PDI) by DLS, their surface charge by electrophoretic light scattering (ELS), and their composition by ^1^H NMR. CSL was incorporated (0, 20, 50 mol %) into POPC large unilamellar vesicles (LUV) or into PDMS_36_-*b*-PEO_23_ large hybrid unilamellar vesicles (LHUV) formed by the hydration method (Table 1). Their non-switchable counterparts (POPC only and PDMS_36_-*b*-PEO_23_:POPC 80:20 and 50:50 mol %) were prepared in a similar fashion and used as control throughout the experiments (Table 1).

The ratios of ^1^H NMR aromatic peaks of CSL and methyl of POPC (CSL/POPC) on the one hand, and CSL and methyl of PDMS_36_-*b*-PEO_23_ (CSL/ PDMS_36_-*b*-PEO_23_) on the other hand, were used to determine the actual amount of CSL after preparation in LUV and LHUV, respectively (Figures S1–S4, Supplementary Materials). The values, close to the initial feeding ratio, indicate that the switchable lipid was successfully incorporated into LUV and LHUV. After extrusion using a 1 µm size filter, surprisingly, the vesicles exhibited a submicron size, which was characteristic of LUV and LHUV [21]. The incorporation of CSL into LUV reversed the ζ-potential, which was due to the cationic character of CSL. The ζ-potential varied according to the CSL amount within LUV or LHUV, accounting for the incorporation of CSL into the membranes. Regarding the hydrodynamic diameter, a strong decrease is observed upon incorporation of CSL into LUV, while the evolution is less clear for LHUV and depends on the amount of CSL incorporated.

### 3.2. Acid-related Morphological Modifications

#### 3.2.1. DLS and ζ-measurements

**Figure 1:**
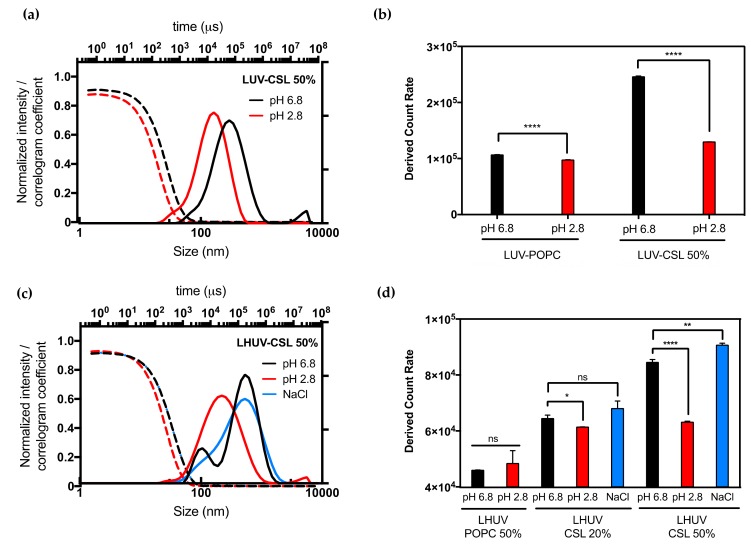
DLS and ζ-potential measurements of LUV and LHUV at pH 6.8 and 2.8. (**a)** Size distribution and correlogram of LUV-CSL 50%; (**b)** Derived count rate and ζ-potential of both LUV-POPC and LUV-CSL 50% at both pHs; (**c)** Size distribution and correlogram of LHUV-CSL 50% at both pHs and after NaCl addition; (**d**) Derived count rate and zeta potential of LHUV-POPC 50%, LHUV-CSL 20% and 50% at both pHs and after NaCl addition. Data represent the mean ±SD (*n* = 3). Student’s *t*-test, where * (*p* < 0.05); ** (*p* < 0.005), **** (*p* < 0.0001) and ns (not statistically significant).

CSL have been reported to undergo a conformational change upon acidification (predicted pK_a_ ≈ 5.39), destabilizing liposomes membrane and promoting the fast cytosolic delivery of siRNA if incorporated at 50 mol % [16]. In this study, the hydrodynamic size distribution and the total scattered intensity (derived count rate (DCR)) were examined before and after the global decrease of pH from 6.8 to 2.8 (Figure 1, Figure S5 and Table S1, Supplementary Materials).

The presence of CSL strongly impacted the properties of both LUV and LHUV as compared to non-responsive vesicles. Upon acidification, LUV-CSL 50% became smaller (from 245 to 126 nm, Table S1) and presented a 2-fold decrease in the derived count rate whilst exhibiting a suitable correlogram profile (Figure 1a,b). In comparison, LUV-POPC did not show any significant change upon acidification (Figure 1b, Figure S5 and Table S1, Supplementary Materials). This observation confirms the presence of CSL into the lipid bilayer and suggests dynamic changes, which is in agreement with previous observations [16]. Interestingly, similar behavior was observed for hybrid vesicles. Acidification significantly decreased the LHUV-CSL size (Figure 1c for LHUV-CSL 50% and Figure S5 for LHUV-CSL 20%) and DCR (Figure 1d and Table S1, Supplementary Materials). The more CSL incorporated into the LHUV, the more marked these effects (Figure 1d). In comparison, LHUV-POPC 20% was not impacted by acidification, neither in size (Figure S5b, Supplementary Materials) nor DCR (Figure 1d). According to DLS measurement, the size distribution is slightly modified with an additional population at slightly higher size for LHUV-POPC 50% (Figure S5c and Table S1, Supplementary Materials), although the DCR remained unchanged (Figure 1d). It has to be noted that the decreased size and DCR in CSL-containing LHUV were not caused by osmotic changes, as the addition of same volume of NaCl at similar molarity did not impact the hydrodynamic diameter and only slightly impacted the DCR at the highest CSL concentration (Figure 1d, and Table S1, Supplementary Materials). Altogether, those results attest that LUV-CSL and LHUV-CSL exhibit pH-triggered changes, which are due to the presence of a switchable lipid embedded in the vesicles’ membrane.

#### 3.2.2. Confocal Observations 

We further investigated the pH-triggered modifications in membranes bearing switchable lipid by macroscopic observations using confocal microscopy. We incorporated CSL into giant unilamellar vesicles (GUV-CSL) or giant hybrid unilamellar vesicles (GHUV-CSL) prepared by electroformation, since their micrometric size is better adapted to confocal microscopy [20]. Their morphological alterations upon global decrease of pH was experimentally examined under confocal microscopy (Figures S6–S9, Supplementary Materials) and posteriorly categorized as vesicles with inward or outward structures (tubular protrusions, membrane-attached aggregation), internalized vesicles, or membrane fluctuation (non-round shaped vesicles) [23] and assembled in a table with values presented as the relative percentage of total vesicles counted (Table 2). As previously, non-switchable giant unilamellar vesicles (GUV-POPC) or giant hybrid unilamellar vesicles (GHUV-POPC) were used as control. It has to be noted that we failed to harvest switchable GHUV containing 50% of CSL after 45 min of electroformation. Therefore, we assessed the acid-induced modifications on GUV-POPC, GUV-CSL 20% (Figure S6, Supplementary Materials), and GUV-CSL 50% (Figure S7, Supplementary Materials), GHUV-CSL 20% (Figures S8–S9, Supplementary Materials) and GHUV-POPC 20% (Figure S8, Supplementary Materials).

The most remarkable observation concerned outward structures after acidification. GUV-CSL 20% and GUV-CSL 50% vesicles responded to acidification by projecting outward tubular protrusions, since 20% and 14% of the vesicles analyzed presented such structures, respectively (Table 2). Membrane-derived structures pointing outward are commonly referred as positive curvatures [23], whilst negative curvature denotates membrane-arisen structures pointing inward. In the images, we could observe positive membrane curvatures in GUV-CSL 20% and 50% as a result of treatment with HCl but not NaCl (Table 2, Figures S6 and S7, Supplementary Materials). Nevertheless, it is difficult to correlate the number of outward structures to the proportion of CSL in the lipid composition, since our preparation method does not guarantee the incorporation of similar amount of lipids in each vesicle.

Interestingly, this phenomenon was concomitant with a relative decrease in membrane fluctuation (Table 2), which could be associated to a transition from irregular shaped-vesicles toward more spherical shaped-vesicles after acid treatment (Figures S6 and S7, Supplementary Materials). Those effects were not observed in non-responsive vesicles, which did not show any substantial changes upon acidification (GUV-POPC, Table 2). Here again, these effects were not due to osmotic shock, since NaCl treatment did not impact CSL-GUV morphology (Table 2, Figure S6, Supplementary Materials). Finally, in HCl-treated GUV-CSL 50%, the number of inward vesicles increased after acidification. This could be attributed to the further rearrangement of irregular-shaped vesicles, dividing into two daughter vesicles, as shown in Video S1 (Centre left, frame 1 to 8, Figure S7 and Video S1, Supplementary Materials).

Regarding the hybrid polymer/lipid structures, similar observations could be drawn, although a different morphology was observed. Whilst GHUV-POPC 20% exhibited spherical and isolated vesicles in agreement with previous reports [20], the presence of CSL in the membrane resulted in aggregated multilamellar structures (Figure S8, Supplementary Materials). Interestingly, the dual labeling of lipids (rhodamine-PE) and polymers (PDMS-NBD) indicated that the polymers and lipids were mixed homogeneously within the membrane (Figure S8, Supplementary Materials). Quantitatively, 28% of GHUV-CSL 20% displayed inward structures and 37% of the population had internalized vesicles under confocal observation, but no outward protruding tubes or membrane fluctuation were observed before acidification. After a global decrease of pH, up to 14% of GHUV-CSL 20% projected outward tubes, which was in a similar fashion than switchable GUV (Table 2, Figure S8, Supplementary Materials). Such quantitative observations, as well as qualitative observations (Figure S9 and Video S2, Supplementary Materials), demonstrate that the presence of CSL triggered dynamic morphological changes, which were mainly observed as outward protrusions, regardless of the membrane’s composition. For GHUV-CSL 20%, this process was accompanied by an increase in the number of fluctuating non-rounded shaped vesicles (from 0 to 7%) and a reduction of the inward structures (Table 2). Furthermore, pH-triggered membrane distortions, leading to hybrid vesicles fission, was another similarity between CSL-bearing GUV and GHUV (GHUV-CSL 20%, frames 1–8, central left vesicle, Figure S9 and Video S2, Supplementary Materials). 

### 3.3. Study of the pH-Triggered Membrane Permeability to GUV and GHUV

#### 3.3.1. pH-Triggered Calcein Release from GUV

**Figure 2:**
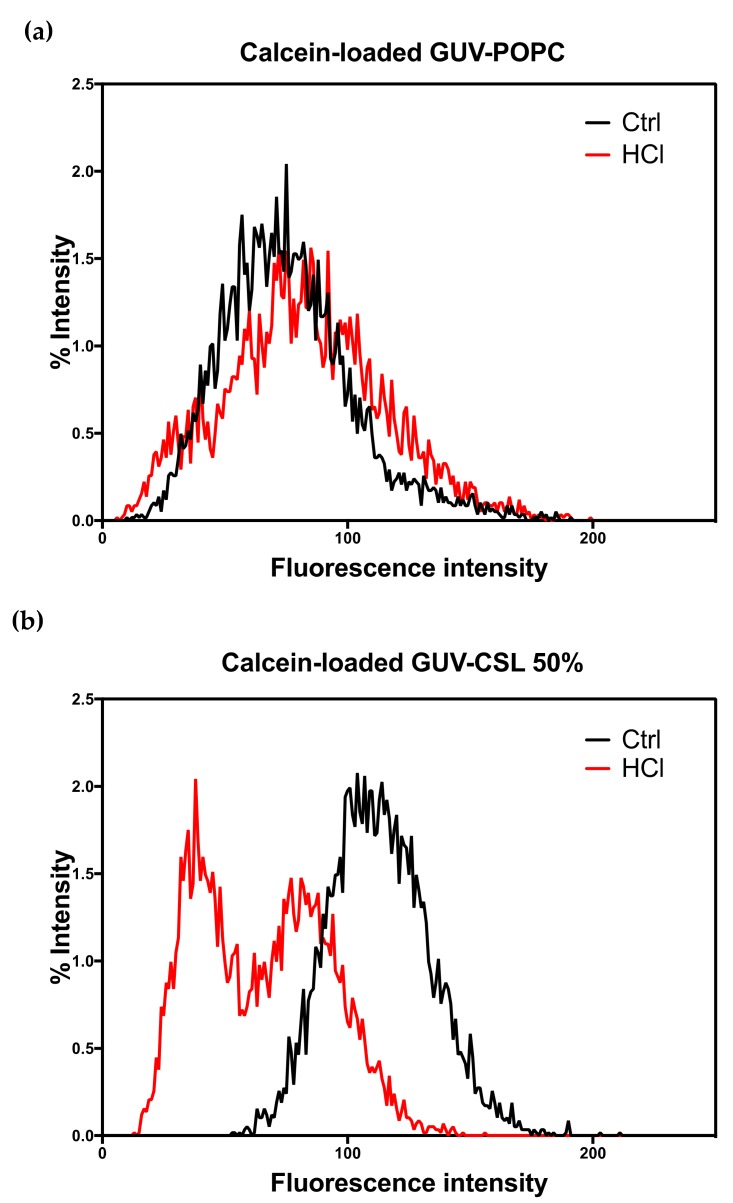
Overall calcein fluorescence intensity in GUVs before and after acidification (pH 6.8 and 4.8). (**a**) Calcein loaded GUV-POPC and (**b**) calcein-loaded GUV-CSL 50%.

Since CSL provided membrane modifications to both lipid-based and hybrid lipid/polymer-based vesicles, we wondered if this behavior could result in pH-triggered permeability and/or drug release ability. Calcein (10 μM) was successfully encapsulated into GUV-CSL 50% and GUV-POPC 50% using the electroformation method. GUV-CSL 20% were not studied since the morphological changes were less significant than for GUV-CSL 50% (Table 2). Calcein was homogenously distributed in the core of GUV-POPC vesicles (Figure S10, Supplementary Materials). However, calcein was not evenly encapsulated into GUV-CSL 50%, which exhibited aggregation and calcein distribution within the core and the membrane of the vesicles (Figure S11, Supplementary Materials). The permeability of calcein-loaded GUV was monitored by confocal microscopy upon acid treatment (see Section 2.5). The overall fluorescence intensity of vesicles was quantified before and after acidification and reported as the percentage of particles exhibiting a specific fluorescence intensity (Figure 2). 

Acidification did not change the morphology (Figure S10, Supplementary Materials) nor the fluorescence intensity (Figure 2a) of the GUV-POPC vesicles. Conversely, GUV-CSL 50% lost their fluorescent content (Figure 2b), demonstrating either the release of calcein or the acidification of the internal cavity of the vesicle, as the fluorescence emission of calcein is pH-sensitive (Figure S11, Supplementary Materials). These results confirm the pH-dependent permeability of the membrane reported for switchable lipids previously [14]. In addition, the time course of these experiments (analysis after 2 min acidification) agrees with the fast conformational switch of the lipids [14]. Remarkably, an uneven distribution of calcein intensity inside GUV-CSL 50% population was observed after acidification (Figure 2). This suggests that all the vesicles did not incorporate the same amount of CSL, since we previously reported that the magnitude of release was related to the proportion of CSL in the lipid composition [14]. 

#### 3.3.2. pH-Triggered Calcein Release from GHUV

**Figure 3:**
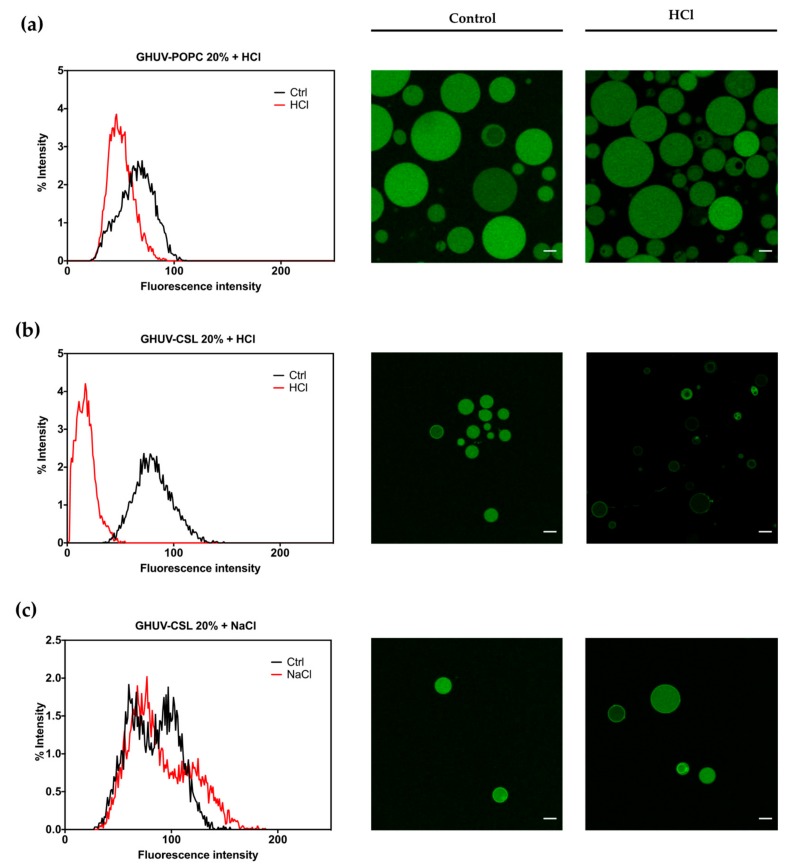
Overall calcein fluorescence intensity distribution in GHUVs before and after acidification. (**a**) Overall intensity in GHUV-POPC 20% followed by confocal pictures before and after acidification; (**b)** overall intensity of loaded-calcein in GHUV-CSL3 20% followed by representative pictures before and after acidification or (**c)** NaCl treatment. Scale bar: 10 μm. Calcein filter.

The impact of CSL incorporation on the behavior of hybrid lipid/polymer vesicles was monitored the same way. Calcein was successfully incorporated into all hybrid lipid/polymer vesicles, yielding homogeneous spherical vesicles, even for GHUV-CSL 50% (Figure S12, Supplementary Materials), which were not harvested without calcein. GHUV-CSL 20% were also much less aggregated and more unilamellar than their empty counterparts (compare Figure S8 and Figure 3b).

A global decrease of pH slightly impacted the intensity of GHUV-POPC 20% vesicles but not the regular round morphology (Figure 3a). In contrast, GHUV-CSL 20% drastically lost their fluorescent content in a few minutes, resulting in mostly empty vesicles (Figure 3b). Video recording demonstrated that the rate of the fluorescence decrease could vary between the vesicles as the CSL content is not controlled from a vesicle to another (Video S3, Supplementary Materials). As previously checked, this phenomenon was not due to osmotic shock, since NaCl treatment did not trigger any release from the GHUV-CSL 20% (Figure 3c). Importantly, significant morphological changes were observed in GHUV-CSL 20% and 50% (Figure 3b and Figure S12, respectively). Protruding and non-rounded shaped fluctuating vesicles (Figure 3b and Video S3, Supplementary Materials) were observed at acidic pH, consistently with unloaded GHUV-CSL (Figure S9). In the case of calcein-loaded GHUV-CSL 50%, only a few aggregated vesicles were visualized after HCl treatment (Figure S12, Supplementary Materials), as the hybrid system was disrupted with global acidification (Video S4, Supplementary Materials).

## 4. Discussion

Molecular tweezers have been recently explored for pharmaceutical applications [24]. Their defined molecular structure allows a fine-tuned control over their conformation, which can be monitored by external stimuli, such as pH, ions, or light [25]. We have previously developed switchable lipids, which exploit a pH-triggered conformational change in order to destabilize a lipid membrane and release hydrophilic drugs and oligonucleotides in the cytoplasm of cells [14,16]. Although the microscopic mechanism of membrane destabilization by CSL is not yet elucidated, we demonstrated that fusion occurred in a pH-responsive fashion and could explain endosomal escape [16]. In this study, we investigated further the biophysical behavior of the lipid membrane upon acidification in order to understand deeper the membrane deformation upon acidification. Although different types of vesicles were examined (LUV and GUV composed of POPC and CSL, on the one hand, and hybrid polymer lipid, LHUV and GHUV composed of PDMS-*b*-PEO diblock copolymer, POPC and CSL, on the other hand), several common features were observed and could be attributed to the presence of the switchable lipid. Firstly, the pH-triggered increase of permeability visualized by calcein fluorescence drop confirmed the previous fluorescence studies of pH-responsive sulforhodamine B leakage from switchable liposomes [14]. In the latter study, 88% of the content was released in less than 15 min. The amplitude of release was related to the amount of the switchable lipid in the lipid composition and to the pH. In addition, the confocal live observations were consistent with a fast responding system (less than 15 min), matching the time course of endosomal maturation [19]. Secondly, we show for the first time that CSL-containing LUV undergo a size change (Table S1, Supplementary Materials) under acidic treatment and that CSL-containing GUV present morphological alterations (Figure S6 and Table 2. Therefore, this study brings additional evidence that pH-triggered macroscopic changes of the lipid membranes are due to the conformational switch of the CSL. Remarkably, the difference between neutral and acidic conditions demonstrates the pH selectivity, which ensures biocompatibility with biological membranes at pH 7.4.

Then, such properties were exploited for hybrid lipid/polymer membranes in order to provide pH-responsive permeability to polymersomes. Hybrid lipid/polymer vesicles have been designed with the idea of overcoming the drawbacks of each component (leakage and mechanical instability for liposomes, low permeability for polymersomes) and combining the benefits of each component (biocompatibility and permeability of liposomes, toughness of polymersomes) [5,26]. Some studies comment about their potential use [27], as nano/micro reactors [28] and/or as drug delivery agents [29] where membrane permeability is of prime importance. Without stimuli-responsive properties, payload release is only achieved by passive diffusion. In this study, we selected PDMS-*b*-PEO diblock copolymers, as they are able to form hybrid vesicles in association with POPC [20]. We used lipid/polymer composition leading to a homogenous phase distribution and the pH-sensitive properties were maintained in GHUV, demonstrating the potential of the switchable lipid.

Macroscopically, GUV-CSL 50% and GHUV-CSL 20% shared a remarkable feature when submitted to HCl treatment: both lipid and hybrid polymer/lipid vesicles underwent morphological changes after global acidification (Table 2, Figure S6 and S8 Supplementary Materials). In particular, the observation of outward structures upon acidification were specific to CSL-containing vesicles, since POPC-containing vesicles did not exhibit such deformations, and they were not due to osmotic changes. Another interesting observation shared among minimal membrane system and hybrid polymer/lipid vesicles was intense membrane fluctuation leading to vesicles fission (Figures S7 and S9, Supplementary Materials). Altogether, the morphological changes observed in this study at a nanometer (LHUV) or micrometer scale (GHUV) are due to the presence of the switchable lipid, which is able to destabilize polymer membranes, and these are reported to be much stiffer than lipid membranes [4,30,31]. To our knowledge, the morphological changes reported here are the first report of pH-sensitive lipid/polymer hybrid vesicles (LHUV or GHUV).

## 5. Conclusions

In this study, we show how a cationic switchable lipid (CSL) impacts the membrane dynamics of a lipid or hybrid lipid/polymer membrane in a pH-responsive manner. At a nanometer scale (LUV and LHUV), the incorporation of CSL resulted in decreased size and count rate, which was not observed for non-responsive vesicles. At a micrometer scale (GUV and GHUV), CSL incorporation resulted in pH-triggered membrane morphological changes and increased membrane permeability. This study gives additional insight to the biological behavior of CSL-based lipid nanoparticles previously reported [16] and open new perspectives in hybrid lipid/polymer vesicle design [5]. In future studies, it would be important to screen different polymers or lipid/polymer blends to investigate the possibility of domains formation, which could lead to a pH-responsive gate in synthetic vesicles. In this case, we need to further develop a fluorescent-tagged switchable lipid to address their distribution within lipid and hybrids polymer/lipid membranes. Moreover, anisotropic NMR could unveil whether the tweezer-like structure arisen from pH-triggered conformational change are responsible for a bilayer incompatible polymorphism [32].

## Figures and Tables

**Table 1:** Physicochemical properties of large unilamellar vesicles and hybrid vesicles (LUVs and LHUVs). CSL: cationic switchable lipid, PDMS_36_-b-PEO_23_: poly(dimethylsiloxane)-*block*-poly(ethylene oxide) backbone, POPC: 1-Palmitoyl-2-oleoyl-sn-glycero-3-phosphocholine.

Name	Composition (% *w/w*)	Measured Molar Ratio (^1^H NMR)	Hydrodynamic Diameter (nm)	PDI	ζ Potential (mV)
**LUV-POPC**	POPC	n/a	687± 6	0.254	−26 ± 1
**LUV-CSL**	POPC:CSL 80:20	80:20	101 ± 24	0.279	+29 ± 1
	POPC:CSL 50:50	54:46	245 ± 3	0.338	+38 ± 1
**LHUV-POPC**	PDMS_36_-*b*-PEO_23_:POPC 80:20	N/D	234 ± 0	0.339	−14 ± 1
	PDMS_36_-*b*-PEO_23_^:^POPC 50:50	N/D	165 ± 9	0.290	−8 ± 1
**LHUV-CSL**	PDMS_36_-*b*-PEO_23_:CSL 80:20	83:17	228 ± 2	0.354	+31 ± 1
	PDMS_36_-*b*-PEO_23_:CSL 50:50	51:49	320 ± 1	0.340	+35 ± 1

N/D: not determined; n/a: not applicable.

**Table 2:** Morphological changes in giant unilamellar lipid vesicles (GUV) and giant hybrid polymer/lipid unilamellar vesicles (GHUV) upon HCl or NaCl treatment.

	Number of Vesicles Analyzed	Vesicles with Inward Structures^1^ 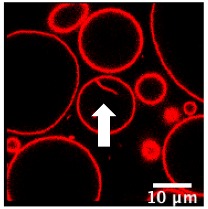	Internalized Vesicles 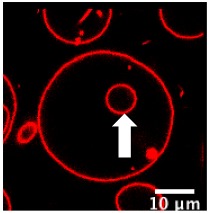	Vesicles with Outward Structures ^1^ 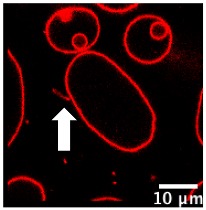	Membrane Fluctuation ^2^ 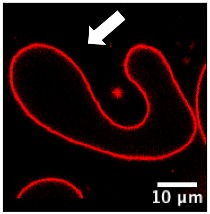
GUV-POPC	64	15%	5%	1.5%	0
GUV-POPC+ NaCl	77	18%	5%	6.5%	0
GUV-POPC + HCl	79	18%	4%	1%	0
GUV-CSL 20%	125	11%	13%	3%	16%
GUV-CSL 20% + HCl	130	11%	16%	20%	4.6%
GUV-CSL 50%	140	9%	6%	6%	9%
GUV-CSL 50% +NaCl	254	6%	6%	2%	8%
GUV-CSL 50%+ HCl	208	14.5%	7%	14%	4%
GHUV-POPC 20%	135	14%	28%	4%	0
GHUV-POPC 20% + HCl	183	11.5%	13%	2%	0
GHUV-CSL 20%	43	28%	37%	0	0
GHUV-CSL 20% + HCl	112	11%	40%	14%	7%

^1^ nanotube, aggregations; ^2^ fluctuating non-rounded vesicles.

## Supplementary Materials

The following are available online at https://www.mdpi.com/2073-4360/12/3/637/s1, Figure S1: ^1^H NMR of GUV-CSL 20% in CDCl_3_, Figure S2: ^1^H NMR GUV-CSL 50% in CDCl_3_, Figure S3: ^1^H NMR GHUV-CSL 20% in CDCl_3_, Figure S4: ^1^H NMR GHUV-CSL 50% in CDCl_3_, Figure S5: Size distribution and correlogram fit of LUV and LHUV at pH 6.8 and 2.8, Figure S6: Morphological changes of GUVs upon HCl or NaCl treatment, Figure S7: Snapshots of morphological changes in GUV-CSL 50% treated with HCl, Figure S8: Morphological changes of GHUVs upon HCl treatment, Figure S9: Snapshots of morphological changes in GHUV-CSL 20% treated with HCl, Figure S10: Permeability of calcein-loaded GUV-POPC 50% treated with HCl, Figure S11: calcein-loaded GUV-CSL 50% treated with HCl, Figure S12: calcein-loaded GHUV-CSL 50% treated with HCl, Table S1: Physicochemical properties of LUVs and LHUVs before and after treatment with HCl or NaCl, video S1: morphological changes in GUV-CSL 50% treated with HCl; video S2: morphological changes in GHUV-CSL 20% treated with HCl; video S3: calcein-loaded GHUV-CSL 20% treated with HCl, video S4: calcein-loaded GHUV-CSL 50% treated with HCl.
