# Supplementary material for: Switchable Lipid Provides pH-Sensitive Properties to Lipid and Hybrid Polymer/Lipid Membranes
Source: Polymers (Basel). 2020 Mar 11;12(3):637. doi: 10.3390/polym12030637 (PMC7183064; doi:10.3390/polym12030637)
Supplement: Supplementary file 1 [file polymers-12-00637-s001.zip › polymers-732202-supplementary materials/Polymers_SI_Passos.pdf]

Supplementary information

Switchable lipid provides pH-sensitive properties to lipid and hybrid polymer/lipid membranes

Victor Passos Gibson [1], Martin Fauquignon [2], Emmanuel Ibarboure [2], Jeanne Leblond Chain [1,3,*] and Jean-François Le Meins [2,*]

[1] Gene Delivery Laboratory, Faculty of pharmacy, University of Montréal, Montréal, QC, Canada; victor.passos.gibson@umontreal.ca
[2] Laboratoire de Chimie des Polymères Organiques LCPO Université de Bordeaux CNRS Bordeaux INP UMR 562916 Avenue Pey Berland F-33600 Pessac France; lemeins@enscbp.fr
[3] ARNA Laboratory, INSERM U1212, CNRS UMR 5320, University of Bordeaux, Faculty of pharmacy, F-33016 Bordeaux, France. Jeanne.leblond-chain@inserm.fr.
* Correspondence: Jeanne.leblond-chain@inserm.fr; lemeins@enscbp.fr

Content

Figure S1 : ^{1}H NMR of GUV-CSL 20% in $CDCl_3$

Figure S2 : ^{1}H NMR GUV-CSL 50% in $CDCl_3$

Figure S3 : ^{1}H NMR GHUV-CSL 20% in $CDCl_3$

Figure S4 : ^{1}H NMR GHUV-CSL 50% in $CDCl_3$

Figure S5: Size distribution and correlogram fit of LUV and LHUV at pH 6.8 and 2.8

Table S1. Physico-chemical properties of LUVs and LHUVs before and after treatment with HCl or NaCl.

Figure S6. Morphological changes of GUVs upon HCl or NaCl treatment.

Figure S7. Snapshots of morphological changes in GUV-CSL 50% treated with HCl.

Figure S8. Morphological changes of GHUVs upon HCl treatment.

Figure S9. Snapshots of morphological changes in GHUV-CSL 20% treated with HCl.

Figure S10. Permeability of calcein-loaded GUV-POPC 50% treated with HCl.

Figure S11. Calcein-loaded GUV-CSL 50% treated with HCl.

Figure S12. Calcein-loaded GHUV-CSL 50% treated with HCl.

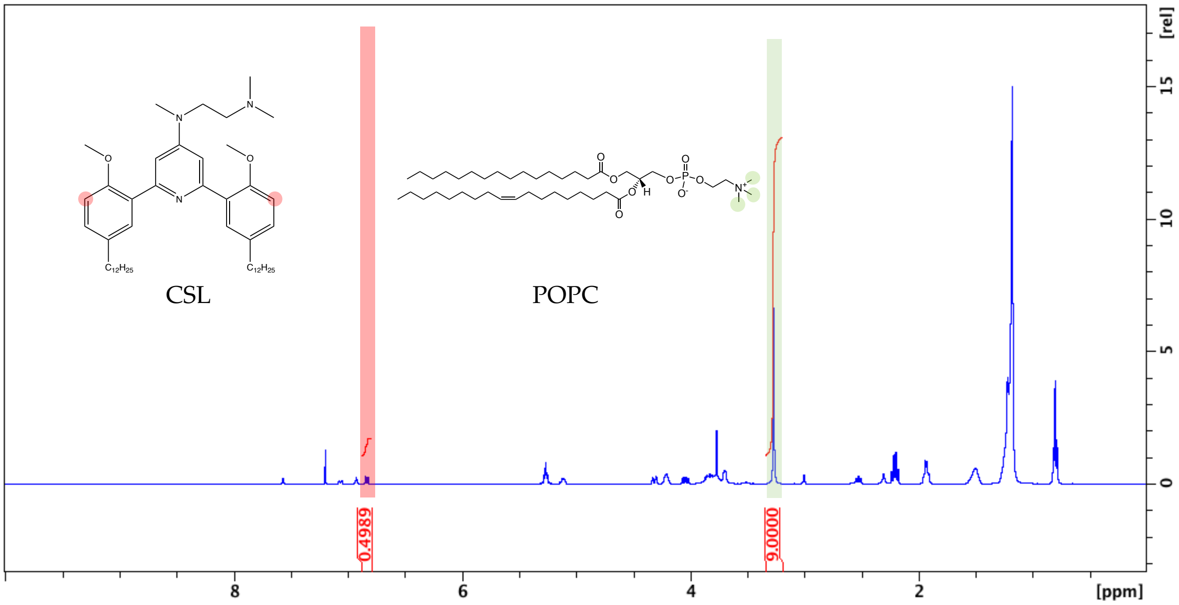

Figure S1. ^{1}H NMR spectrum of GUV-CSL 20% in $CDCl_3$

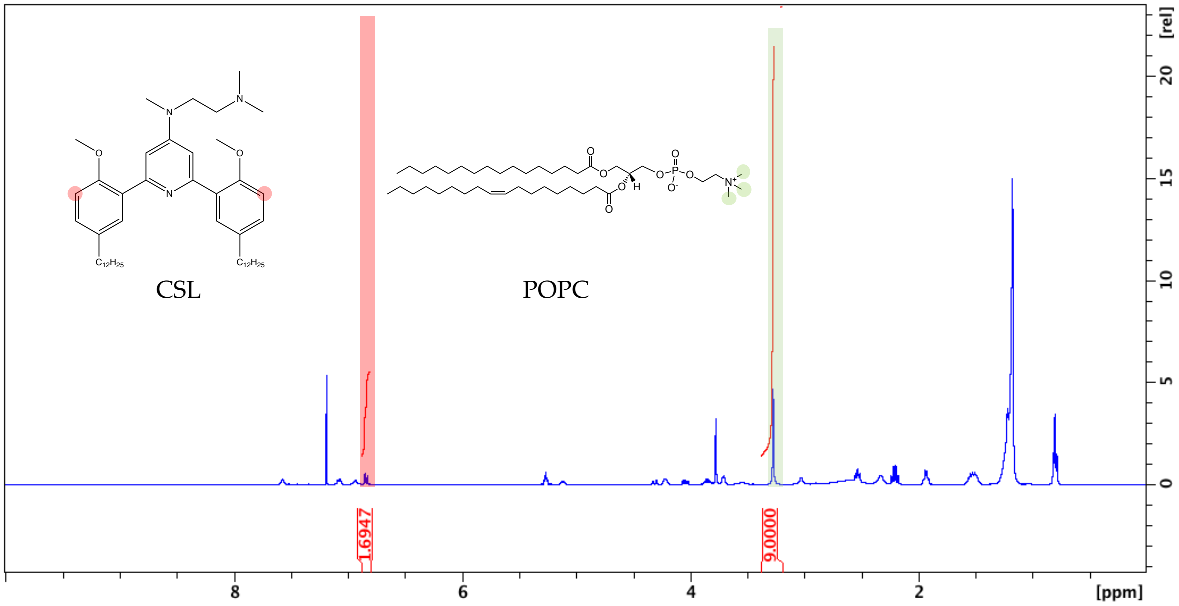

Figure S2. ^{1}H NMR spectrum of GUV-CSL 50% in $CDCl_3$

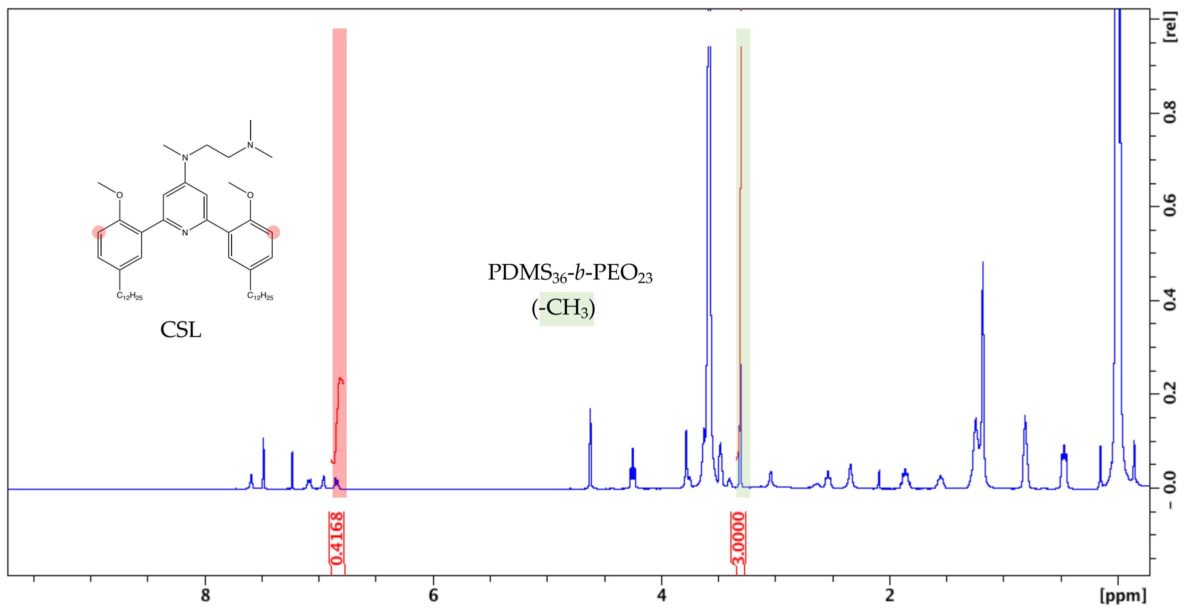

Figure S3. ¹H NMR spectrum of GHUV-CSL 20% in $CDCl_3$

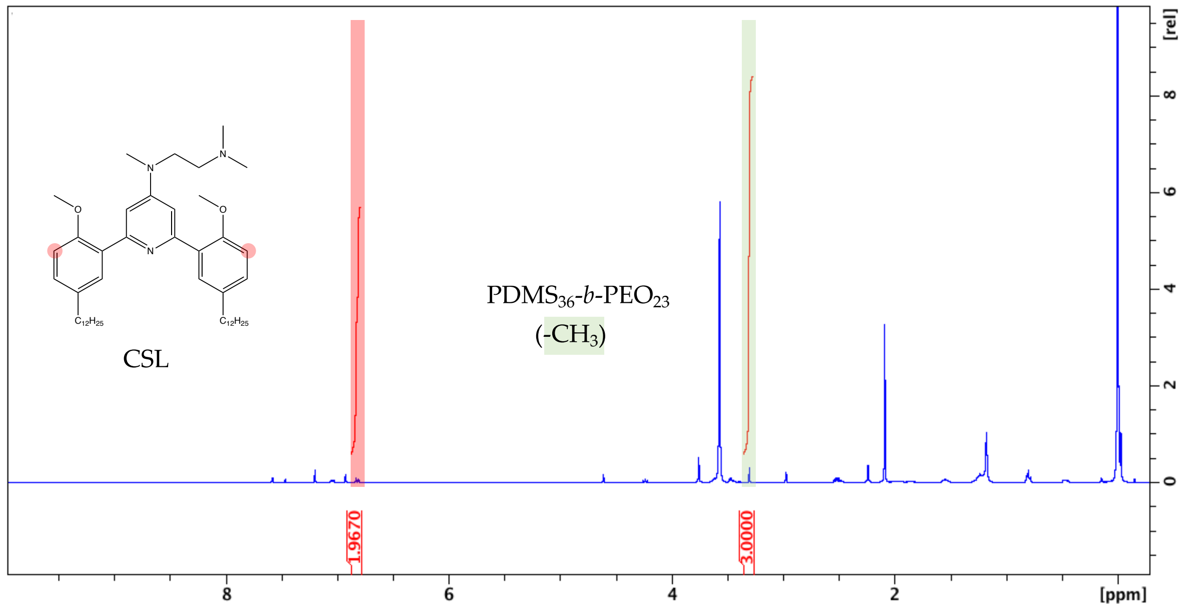

Figure S4. ¹H NMR spectrum of GHUV-CSL 50% in $CDCl_3$

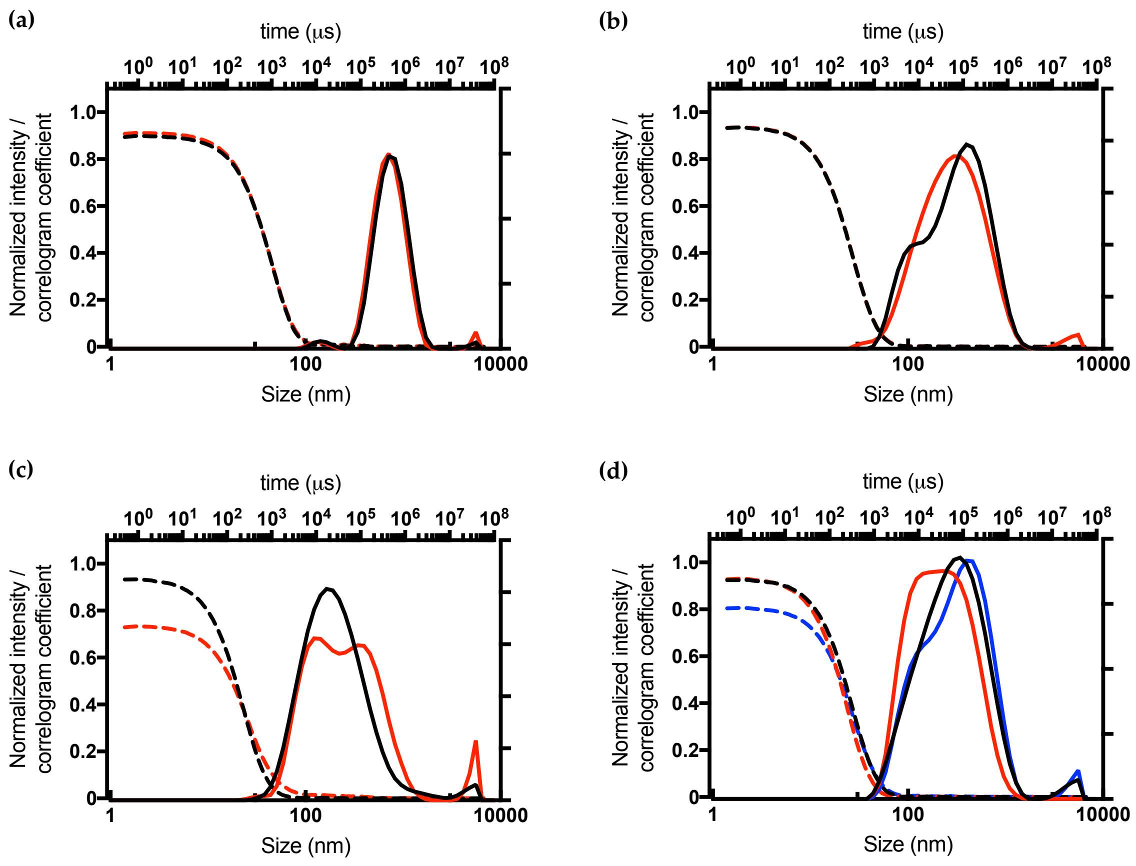

Figure S5. Size distribution and correlogram fit of LUV and LHUV at pH 6.8 and 2.8. (**a**) LUV-POPC, (**b**) LHUV-POPC 20%, (**c**) LHUV-POPC 50%, (**d**) LHUV-CSL 20%. Black line: pH 6.8; red line: pH 2.8; blue line: NaCl.

Table S1. Physico-chemical properties of LUVs and LHUVs before and after treatment with HCl or NaCl.

Name	Composition (% mol)	Hydrodynamic diameter (nm) (PDI)			DCR (Derived count rate)		
		pH 6.8	pH 2.8	NaCl	pH 6.8	pH 2.8	NaCl
LUV-POPC	n/a	687± 6 (0.254)	657 ± 9 (0.336)	ND[1]	1.0 x 10^5 ± 0.6 x 10^3	0.9 x 10^5 ± 0.4 x 10^3	ND
LUV-CSL	POPC:CSL 50:50	245 ± 3 (0.338)	126 ± 1 (0.243)	ND	2.4 x 10^5 ± 1.3 x 10^3	1.2 x 10^5 ± 0.2 x 10^3	ND
LHUV-POPC	PDMS$_{36}$-*b*-PEO$_{23}$: POPC 80:20	234 ± 1 (0.339)	232 ± 3 (0.360)	ND	6.1 x 10^4 ± 0.2 x 10^3	6.1 x 10^4 ± 0.1 x 10^3	ND
	PDMS$_{36}$-*b*-PEO$_{23}$: POPC 50:50	165 ± 2 (0.291)	216 ± 1 (0.514)	ND	4.5 x 10^4 ± 0.1 x 10^3	4.8 x 10^4 ± 3.7 x 10^3	ND
LHUV-CSL	PDMS$_{36}$-*b*-PEO$_{23}$: CSL 80:20	228 ± 2 (0.354)	177 ± 1 (0.250)	242 ± 8	6.4 x 10^4 ± 1.0 x 10^3	6.1 x 10^4 ± 0.07 x 10^3	6.7 x 10^4 ± 2.2 x 103
	PDMS$_{36}$-*b*-PEO$_{23}$: CSL 50:50	320 ± 1 (0.340)	183 ± 1 (0.297)	312 ± 10	8.4 x 10^4 ± 0.8 x 10^3	6.3 x 10^4 ± 0.3 x 10^3	9.0 x 10^4 ± 0.5 x 10^3

[1]Not determined

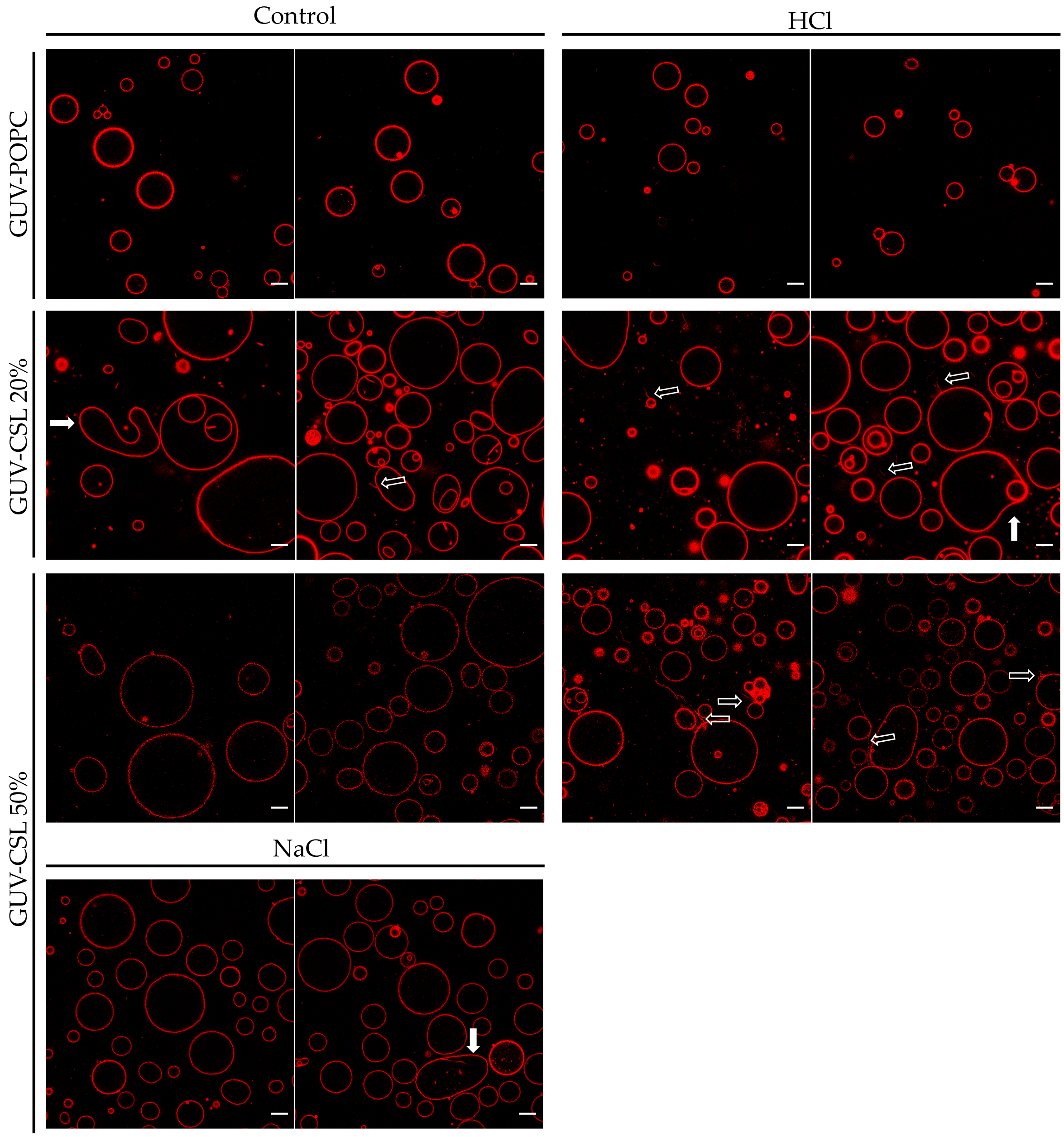

Figure S6. Morphological changes of GUVs upon HCl or NaCl treatment. Open arrows: vesicles with outward projections. Full arrows: membrane fluctuation events. Scale bar: 10 μm.

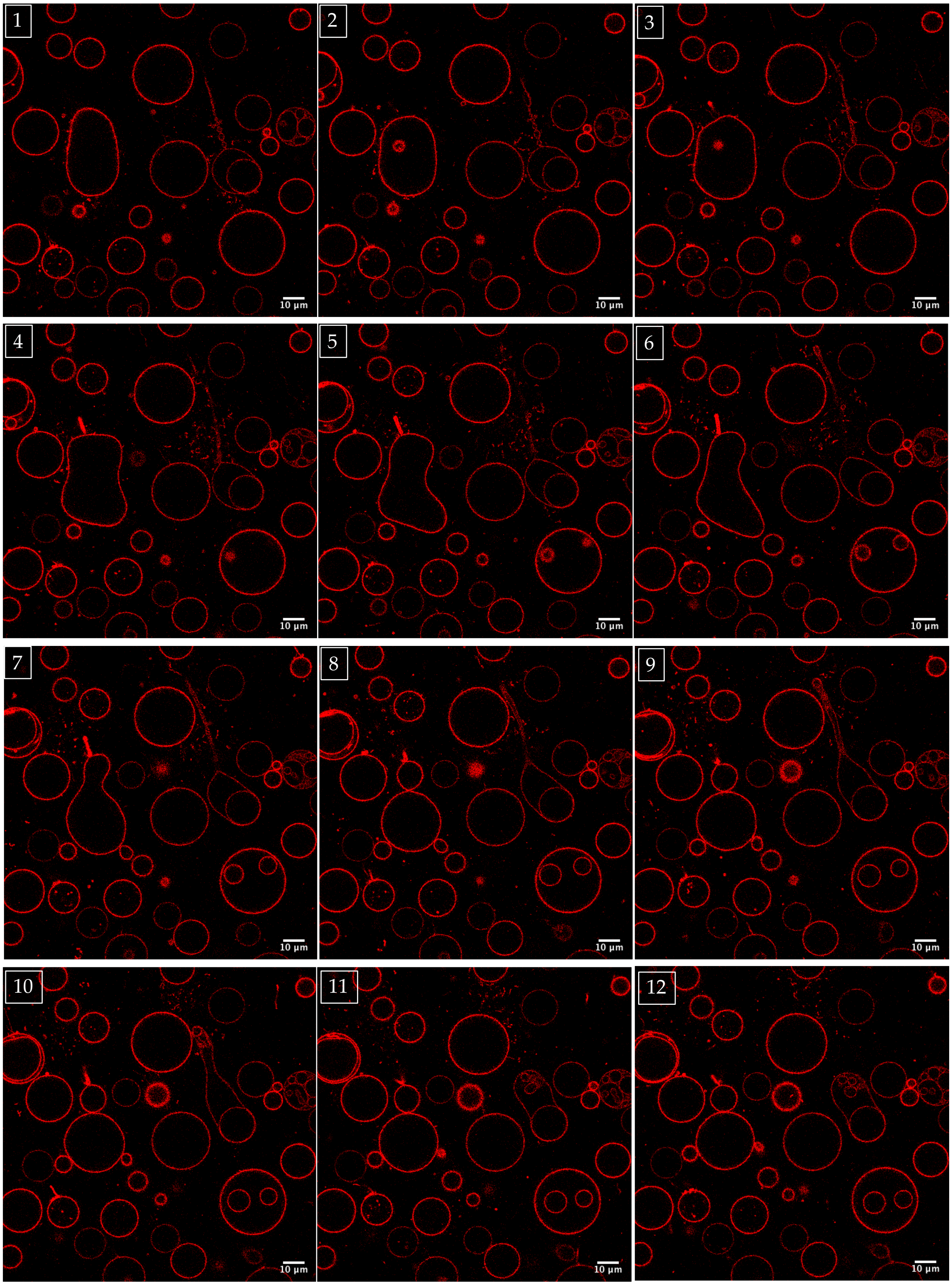

Figure S7. Snapshots of morphological changes in GUV-CSL 50% treated with HCl. White boxes in the upper left corner indicate frame order. Time-lapse between pictures is 0.143 second; total time-lapse is 1.86 seconds. Scale bar: 10 µm.

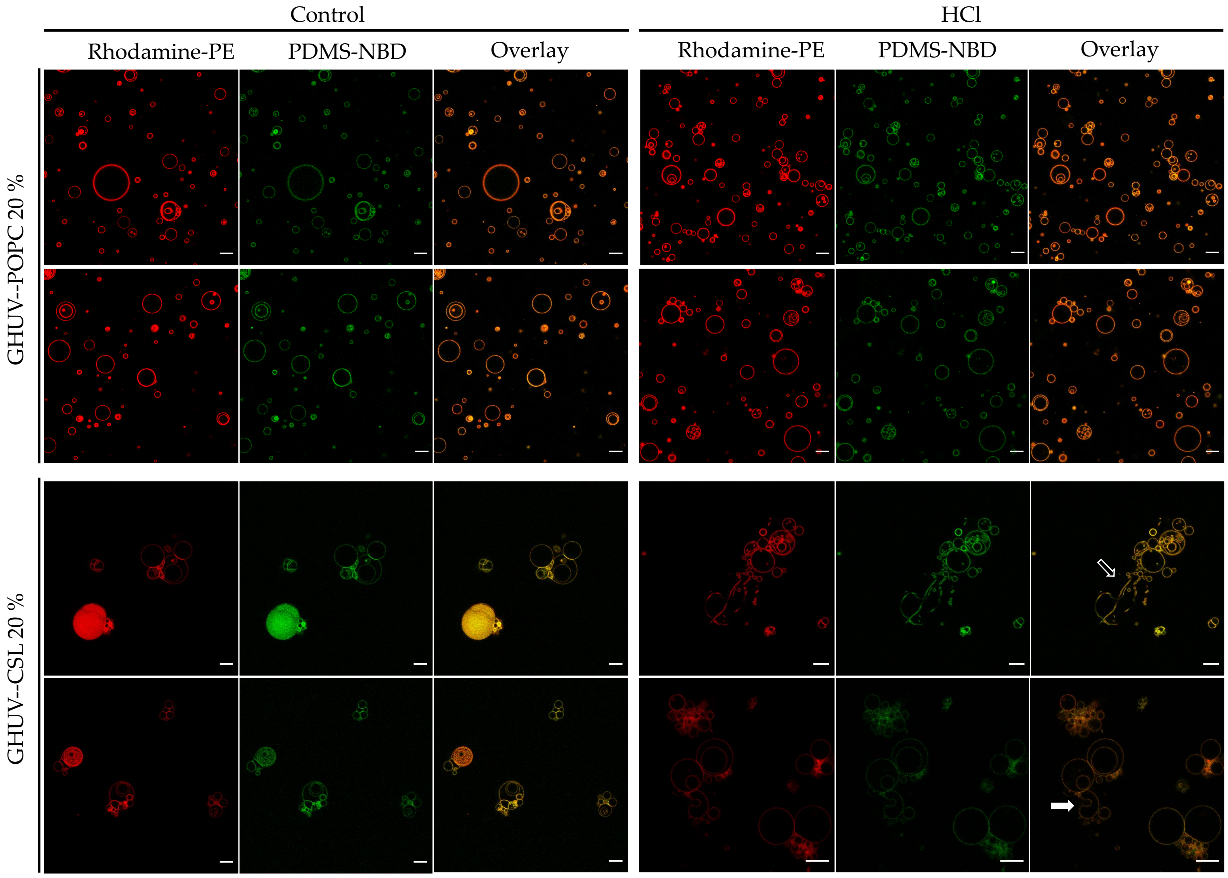

Figure S8. Morphological changes of GHUVs upon HCl treatment. Open arrows: vesicles with outward projections. Full arrows: membrane fluctuation events. Scale bar: 10 μm.

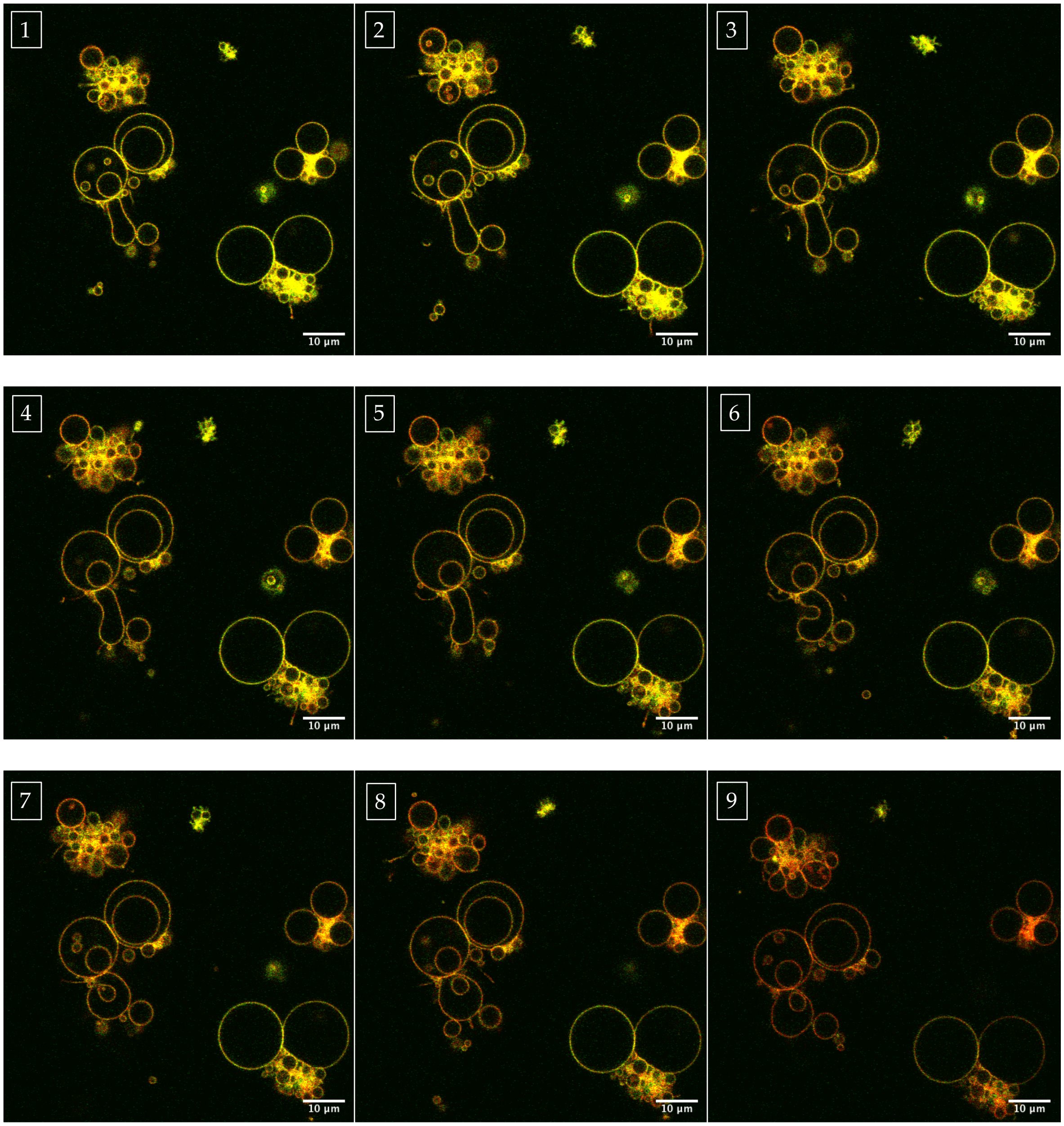

Figure S9. Snapshots of morphological changes in GHUV-CSL 20% treated with HCl. Merged PDMS-NBD and Rhodamine-PE filters. Time-lapse between pictures is 0.694 second; total time-lapse is 6.25 seconds. Scale bar: 10 µm.

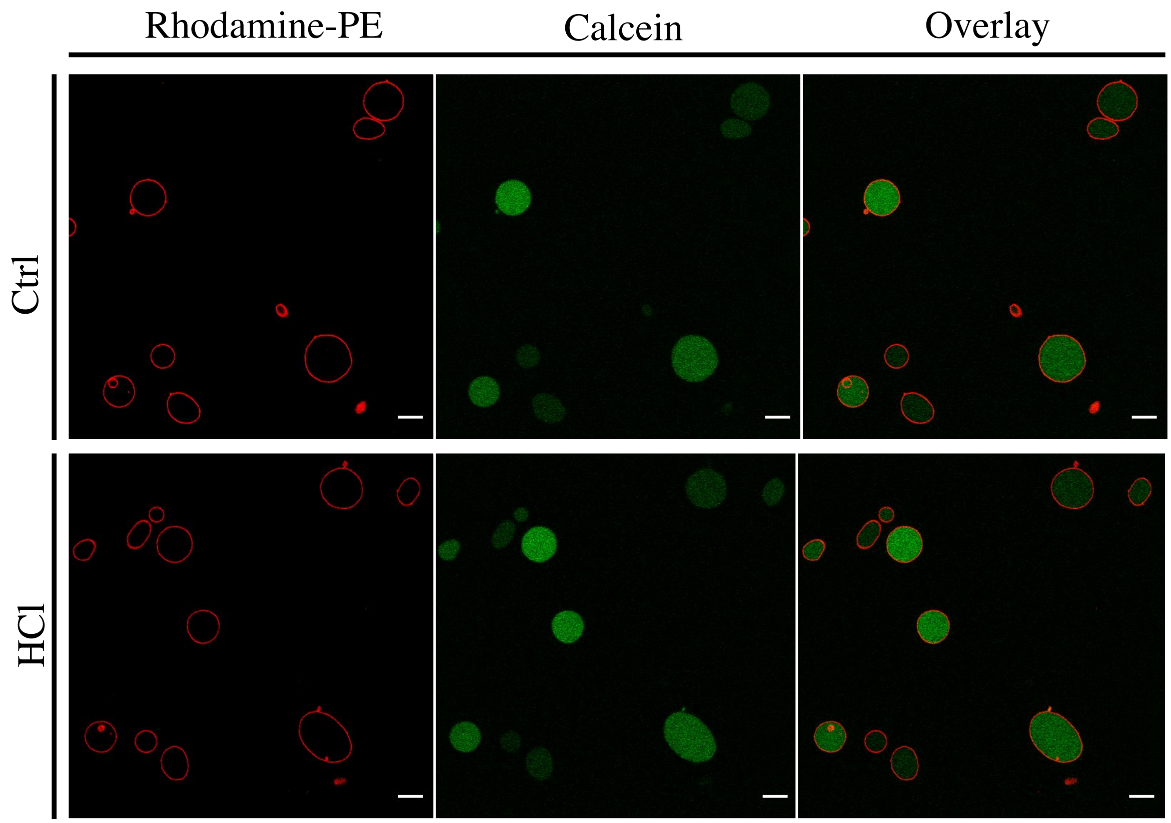

Figure S10. Permeability of calcein-loaded GUV-POPC 50% treated with HCl. Snapshots of morphological changes in GUV-POPC 50% treated with HCl. Rhodamine, calcein and merged filters. Scale bar: 10 µm

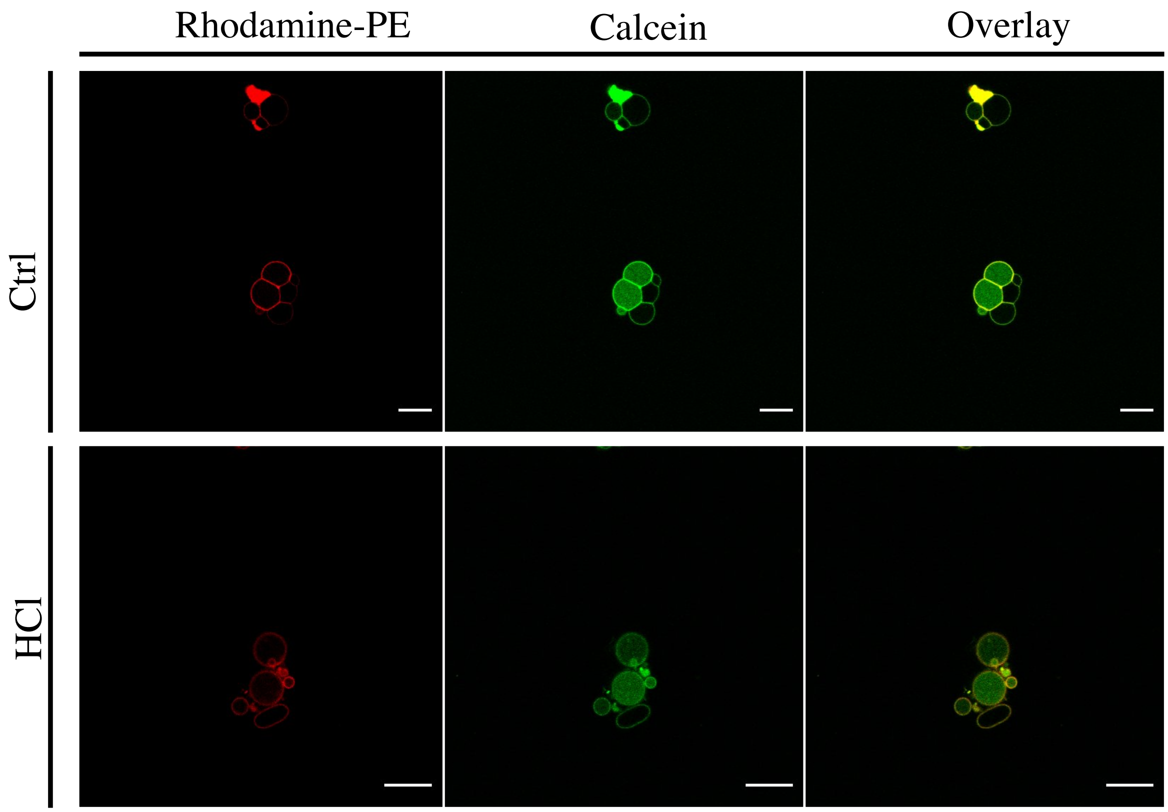

Figure S11. Calcein-loaded GUV-CSL 50% treated with HCl. Rhodamine, calcein and merged filters. Scale bar: 10 μm

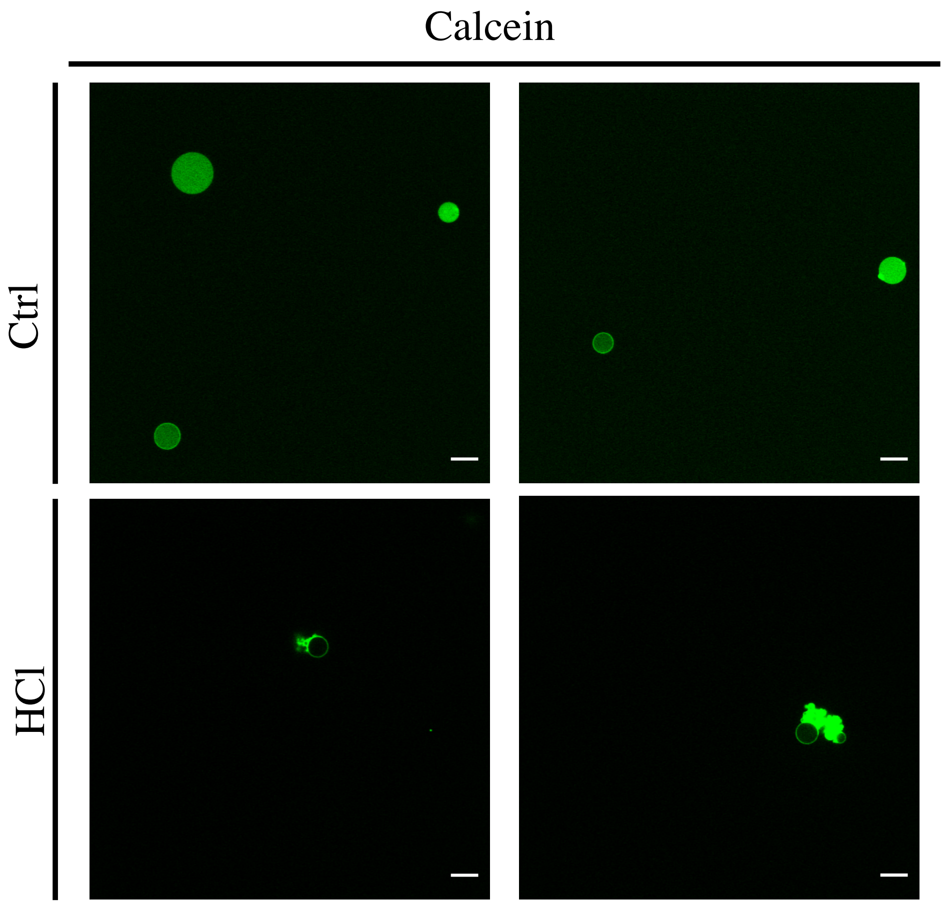

Figure S12. Calcein-loaded GHUV-CSL 50% treated with HCl. Calcein filters. Scalebar: 10 μm.
